# Innate-Adaptive Immune Crosstalk

**DOI:** 10.1155/2015/982465

**Published:** 2015-11-04

**Authors:** Anil Shanker, Menaka C. Thounaojam, Manoj K. Mishra, Mikhail M. Dikov, Roman V. Uzhachenko

**Affiliations:** ^1^Department of Biochemistry and Cancer Biology, School of Medicine, Meharry Medical College, Nashville, TN 37208, USA; ^2^Host-Tumor Interactions Research Program, Vanderbilt-Ingram Cancer Center, Vanderbilt University, Nashville, TN 37232, USA; ^3^Cancer Biology Research and Training Program, Department of Biological Sciences, Alabama State University, Montgomery, AL 36104, USA; ^4^Department of Medicine, James Cancer Center, The Ohio State University, Columbus, OH 43210, USA

The vertebrate immunological defense system relies upon interdependent regulatory interactions between the innate and adaptive immune compartments playing a pivotal role in various physiological and immunopathological conditions. Recent studies have demonstrated that interactions between dendritic cells and NK cells or T cells and NK cells are important for clearing various bacterial and viral infections. The importance of intratumoral crosstalk between T cells and NK cells during their antitumor immune response has also been established for efficient tumor rejection. Our work in mouse tumor models [1, 2] and others work in models of obesity [3–5], atherosclerosis [6], peritonitis [7], viral and bacterial infections [8–10], and intestinal microbiota [11] suggest a much broader bidirectional cooperativity of the adaptive and innate immune functions. It is, thus, imperative to study both these immune compartments as one functional unit. Detailed understanding of the interaction between innate and adaptive immunity can lead to new approaches aimed to improve immunotherapy for various diseases. This special issue consists of 4 review and 5 research articles.

Behçet's disease is an inflammatory disorder characterized by orogenital ulcerations, ocular manifestations, arthritis, and vasculitis. Md. S. Hasan and colleagues reviewed the role of gamma delta (*γδ*) T cells' involvement in Behçet's disease and addressed their potential interactions with neutrophils and monocytes mediated by proinflammatory cytokines TNF*α* and IFN*γ* and the chemokine CXCL8. In another review article, IFN*γ* priming as a mechanism affecting both innate immune cells and effector memory CD4^+^ T cells was reviewed by H. B. da Silva et al. IFN*γ* is the main cytokine produced by effector memory T cells committed for the Th1 phenotype. Compiling available literature on various infectious and chronic diseases, authors make the case that IFN*γ* is an important point of crosstalk between innate and adaptive immunity. The review article by P. de Matos Silva and colleagues provides an update on current therapies based on tolerogenic dendritic cells and their crosstalk with T cells modulated by costimulatory blockers with the aim of reducing transplant rejection. They also highlighted challenges for allograft rejections. They review the successes and failures of clinical trials employing iNKT cell-based immunotherapy and explore the future prospects of using such strategies. A review article by J. B. Altman et al. focused on the role of a specific lineage of NKT cells and their interaction with tumor-associated macrophages influencing tumor microenvironment and antitumor immunity.

The research article collection for this special issue covers 2 clinical and 3 preclinical research articles. M. Wu et al. studied the immunoregulatory effects of human umbilical cord's mesenchymal stem cells (UC-MSCs) on IL-22 production in patients with immune thrombocytopenia (ITP). Herein, authors reported for the first time that UC-MSCs downregulate IL-22 through soluble cellular factors but not PGE2 in ITP patients. The study by M. Lima and colleagues evaluated chemokine receptor expression on normal blood CD56^+^ NK cells in a network with other immune cells. They identify a transitional NK cell population between the CD56^high^ and CD56^low^ NK cell populations, which is CXCR3/CCR5^+^ with intermediate expression levels of CD16, CD62L, CD94, and CD122.

I. J. Kosten et al. studied crosstalk between keratinocytes, fibroblasts, and Langerhans cells and showed that the proinflammatory cytokine, IL-18, and chemokines CCL2, CCL20, and CXCL12 are mostly secreted by skin, when compared with gingiva. Furthermore, CCL27 was primarily secreted by skin whereas CCL28 was mostly released by gingiva. This suggests that the cytokines and chemokines involved in triggering and mediating Langerhans cell migration and the innate immune response are different in skin and gingiva. D. Wang et al. explored potential natural ligands of antitumor monoclonal antibody HAE3 by performing carbohydrate microarrays. Authors demonstrate that HAE3 recognizes a conserved cryptic glycoepitope of blood group precursors, which is nevertheless selectively expressed and surface-exposed in certain human breast cancer cell lines, including some triple-negative ones that lack the estrogen, progesterone, and Her2/neu receptors. Findings by X.-T. Yin et al. indicated that the interaction of Fas with FasL in the cornea restricts the development of recurrent herpetic stromal keratitis (HSK) following herpes simplex virus-1 (HSV) infection of the cornea. Authors demonstrated that infection of the cornea with HSV-1 results in increased functional expression of FasL in ocular tissues and that mice expressing mutations in Fas (lpr) and FasL (gld) display increased recurrent HSK following reactivation than do wild-type mice. This clinical disease is the result of a crosstalk of inflammatory cells, consisting of polymorphonuclear leukocytes, macrophages, and T cells (both CD4^+^ and CD8^+^) that are recruited to the corneas of patients with HSK.

The presented collection of papers further highlights the cross-regulatory interaction between the innate-adaptive immune networks. We hope that these articles will encourage more clinical and basic studies focused on understanding the malleable functional innate-adaptive immune crosstalk by abandoning the rigid taxonomic dichotomy of immunity.
